# Silybin Alleviates Experimental Autoimmune Encephalomyelitis by Suppressing Dendritic Cell Activation and Th17 Cell Differentiation

**DOI:** 10.3389/fneur.2021.659678

**Published:** 2021-09-07

**Authors:** Huan-Li Yang, Xiao-Wu Shi

**Affiliations:** ^1^Xian Yang Central Hospital, Xianyang, China; ^2^Xian Yang Central Blood Station, Xianyang, China

**Keywords:** EAE, multiple sclerosis, silybin, dendritic cell, T cell

## Abstract

Silybin, a peculiar flavonoid compound derived from the fruit and seeds of *Silybum marianum*, exhibits strong anti-inflammatory activities. In the present study, we found that silybin effectively alleviated experimental autoimmune encephalomyelitis (EAE), an animal model of multiple sclerosis (MS), via inhibition of dendritic cell (DC) activation and Th17 cell differentiation. Silybin treatment greatly ameliorated the disease severity and significantly declined inflammation and demyelination of the central nervous system (CNS) of EAE mice. Consistent with the disease development, silybin-treated bone marrow-derived DCs (BM-DCs) exhibited reduced costimulatory molecules (e.g., CD80 and CD86) and MHC II expression. These results demonstrated the distinguished bioactivity of silybin for suppressing DC activation, inhibiting pathogenic Th17 inflammatory cell responses, and, eventually, alleviating EAE severity. Taken together, our results show that silybin has high potential for the development of a novel therapeutic agent for the treatment of autoimmune diseases such as MS.

## Introduction

Multiple sclerosis (MS) is an inflammatory and neurodegenerative demyelinating disease of the central nervous system (CNS). The pathogenesis of MS is multifactorial, involving genetic and environmental elements interacting in complicated ways. The history of MS therapy is a wonderful instance of a successful investigation translated into treatments and enhanced clinical results (1). Experimental autoimmune encephalomyelitis (EAE), as a classic animal model of MS, is widely applied for drug development and discovery. Although the definite pathogenesis has not been illuminated clearly, increasing evidences endorse that MS is an autoimmune disease with irreversible white matter (WM) damage (2, 3). At the early phase of EAE, myelin-specified CD4^+^ T cells, as well as dendritic cells (DCs), B cells, and macrophages, are triggered in the periphery and penetrated the CNS. Among diverse CD4^+^ T-cell subsets, interleukin-17 (IL-17)-positive Th17 cells, which secrete IL-17A, are regarded as the primary effector cells in provoking an inflammatory response in MS/EAE (4). In addition, antigen-presenting cells (APCs) also exert an essential function in MS/EAE by activating naïve T cells, among which DCs are experts at regulating rest T-cell polarization with antigen peptides present (5).

Current clinical treatments for MS possess either insufficient performance or uncertain safety problems. Numerous researches have been devoted to expanding novel therapeutic drugs target Th17 cells without affecting other cells. In the past few years, a lot of immune-modulatory monomers derived from medicinal plants exhibit a tremendous capacity for treating MS/EAE. These small molecule natural compounds present excellent fortune for identifying effective and safe medicine candidates. Silymarin is a peculiar flavonoid compound derived from the fruit and seeds of *Silybum marianum*. It contains a group of flavonolignans, such as silybin A, silybin B, isosilybin A, isosilybin B, silychristin, isosilychristin, silydianin, and taxifolin (6). Silybin, also known as silibinin, is the major biologically active constituent of the silymarin complex (about 70–80%) and is a mixture of silybin A and silybin B. Pharmacological research demonstrated that silybin possesses potent antioxidant, anticarcinogenic, and anti-inflammatory activities (7–9). Moreover, Lee et al. also proved that silybin has excellent therapeutic effects on EAE by inhibiting the polarization of Th1. However, the activity of silybin on Th17 and DC development is still unclear. To tackle these issues, here, we employed the MOG_35−55_-induced autoimmune animal model to explore the therapeutic activities and the underlying mechanism of silybin. The efficacy of silybin made it a potential therapeutic drug for alleviating EAE as well as other autoimmune diseases.

## Materials and Methods

### Experimental Autoimmune Encephalomyelitis Induction and Drug Treatment

Female C57BL/6 mice (8 weeks old) were purchased from the Air Force Medical University (Xi'an, China). All the animal experiments were performed following the Guidelines for Care and Use of Laboratory Animals of Xian Yang Central Hospital and authorized by the Animal Ethics Committee of Xian Yang Central Hospital. The EAE installation procedure was as outlined previously (10). Briefly, mice were subcutaneously immunized at two sites on the back with 200 mg of myelin oligodendrocyte glycoprotein_35−55_ (MOG_35−55_; Genscript, Piscataway, NJ, USA) in 200 μl of emulsion comprising 50% complete Freund's adjuvant with 5 mg/ml of *Mycobacterium tuberculosis* H37Ra (Difco Laboratories, Lawrence, KS, USA). Pertussis toxin (PT) (200 ng/mouse; Sigma-Aldrich, St. Louis, MO, USA) was administrated intraperitoneally (i.p.) to the mice on day 0 and 2 days post-immunization (p.i.). Clinical scores were record daily in a blind manner, according to a 0–5 scale as described previously (11). Accumulative scores of each mouse were calculated by adding scores of the mouse from day 10 to day 30 p.i. Silybin was prepared in dimethyl sulfoxide (DMSO) for stock. Five percent of DMSO was dispersed in phosphate-buffered saline (PBS) designated as the vehicle. Vehicle or silybin (5, 10, and 20 mg/kg) was given by i.p. each day and starting from day 0 p.i., or day 10 p.i. (disease onset, 10 mg/kg), or day 19 (disease peak, 10 mg/kg).

### Histopathology

For immunohistochemistry staining, lumbar spinal cords (SCs) were harvested following EAE mice after PBS perfusion and fixed in 4% paraformaldehyde (PFA) for 1 day 25°C. Samples were plated in paraffin for slide stain with hematoxylin and eosin (H & E) and luxol fast blue (LFB). The slides were sectioned coronally at 5 μm. Sections were evaluated and scored in a blind manner for inflammation and demyelination by following previous methods (12). For inflammation, 0 means none; 1, a few inflammatory cells; 2, organization of perivascular infiltrates; and 3, increasing severity of perivascular cuffing with extension into the adjacent tissue. For demyelination, 0 means none; 1, rare foci; 2, a few demyelination areas; and 3, large (confluent) areas of demyelination.

For immunohistochemistry, SC tissues were fixed with 4% PFA for 24 h and then cryo-protected by 30% sucrose solvent for 72 h. Samples were plated in optimal cutting temperature (OCT) compound (Tissue-Tek, Sakura Finetek, Tokyo, Japan) for frozen sections and then cut coronally in 8-μm sections. Transverse sections of SC were stained with myelin basic protein (MBP). The slides were incubated with primary antibody diluted in blocking buffer overnight at 4°C. The primary antibody used was rabbit anti-MBP (Abcam, Cambridge, UK; ab40390; 1:1,000). Secondary detection was performed with Alexa Fluor-488 secondary antibodies (Jackson ImmunoResearch Laboratories, West Grove, PA, USA; 111-545-144; 1:750) for 1 h. ProLong Diamond Antifade Mountant with DAPI buffer (Thermo Fisher Scientific, Waltham, MA, USA; P36962) was used to mount the slides. Results were visualized by fluorescence microscopy (Nikon DS-Ri2, Nikon, Tokyo, Japan). For the myelinated region calculations, 10 areas in the WM of the lumbar SC were chosen and analyzed by Image-Pro Plus software.

### Mononuclear Cell Preparation

To collect the mononuclear cells (MNCs) from the periphery for flow cytometry analysis, the mice were treated with silybin or vehicle at day 0 and sacrificed at day 21 p.i. Splenocytes were mechanically dissociated through a 70-μm cell strainer (Corning, New York, NY, USA) and reacted with red blood cell (RBC) lysis buffer (BioLegend, San Diego, CA, USA) for 1 min. The cells were then washed with cold PBS before stimulation. The spleen cells were seeded at 1.0 × 10^6^ cells/ml and cultured in triplicates in Roswell Park Memorial Institute (RPMI) 1640 supplemented with 10% fetal bovine serum (FBS) in 24-well plates and pulsed with 25 μg/ml of MOG_35−55_ for 3 days.

To harvest CNS infiltrated MNCs, EAE mice were perfused with 20 ml of PBS to remove blood and collect the penetrated MNCs from CNS. The SCs and brains of each group were dissociated by Neural Tissue Dissociation Kits (Miltenyi Biotec, Bergisch Gladbach, Germany) to digest into cell suspensions and then filtered with a 40-μm cell strainer. MNCs were isolated in a 70%/30% Percoll medium with a 2,000-rpm centrifuge for 20 min. After removal of the myelin debris in the upper layer, the MNCs were harvested from the middle interface to be used in the following experiments (13). The infiltrated MNCs were seeded at 1.0 × 10^6^ cells/ml and were cultured in triplicates in RPMI 1640 supplemented with 10% FBS in 24-well plates and pulsed with 10 μg/ml of MOG_35−55_ for 1 day.

### T-Cell Proliferation

For *ex vivo* proliferation, splenocytes were isolated 21 days p.i. from vehicle- and silybin-treated mice. To test the proliferation efficiency, the cells were treated with or without stimuli [25 μg/ml of MOG_35−55_ or 5 μg/ml of concanavalin A (Con A)]. Cell proliferation was determined by the BrdU-incorporation test using BrdU Cell Proliferation ELISA Kit (Abcam, cat no. ab126556).

### T-Cell Differentiation

Spleen cells were separated from 8-week-old C57BL/6 naïve mice, and single-cell suspensions were obtained following a previously described method (2). Naïve CD4 microbead (Miltenyi Biotec) was applied to purified CD4^+^ T cells. Subsequently, under 72 h of differentiation medium, cells were differentiated and analyzed on BD FACSAria (BD Biosciences, San Jose, CA, USA). In short, the cells were cultivated with anti-CD3ε (1 μg/ml) and anti-CD28 (1 μg/ml) under their differentiating medium. IL-12 (5 ng/ml) and anti-IL-4 (10 μg/ml) were added to prompt polarization into Th1 cells. Anti-IFN-γ (10 μg/ml), IL-2 (10 ng/ml), and IL-4 (10 ng/ml) were added to prompt polarization into Th2 cells. IL-6 (20 ng/ml), TGF-β1 (2 ng/ml), IL-1β (10 ng/ml), anti-IL-4 (10 μg/ml), and anti-IFN-γ (10 μg/ml) were added in Th17 polarization medium. TGF-β1 (2 ng/ml) and IL-2 (10 ng/ml) were added to induce polarization into Treg cells.

### Dendritic Cell Culture and Activation

To obtain the bone marrow-derived DCs (BM-DCs), femurs and tibias were isolated from the naïve C57BL/6 mice (8 weeks), and the cells were flushed out of the bone marrow with a 30-gauge needle. After flow-through by 100-μm cell strainer, the obtained cells were cultivated in RPMI 1640 medium. Cells were cultured for 10 days; and medium is changed every 4 days supplemented with granulocyte/macrophage colony-stimulating factor (GM-CSF; 10 ng/ml) to obtain mature BM-DCs. DCs are cultured in a medium without any cytokine stimulation after 10 days and activated with 100 ng/ml of lipopolysaccharides (LPSs) for the subsequent experiments. Silybin (50 μM) was added to the medium simultaneously (5).

For DC co-cultured with T cells, DCs were cultivated overnight at a density of 1 × 10^4^ cells/ml in 96-well U-bottomed plates in RPMI added with 10% FBS, 2 mM of l-glutamine, 100 ng/ml of LPS with or without silybin (50 μM), and 10 μg/ml of MOG_35−55_ peptide. After 24 h, the DCs were washed, and then aliquots of 1 × 10^5^ cells/ml of naïve T cells were co-cultured with activated DC cells for 3 days and then used for flow cytometry analysis (14).

### Real-Time Quantitative PCR

According to the manufacturer's instructions, RNA was isolated from the RNAprep Pure Tissue Kit (Tiangen Biotech, Beijing, China). Reverse transcription was conducted using the Prime Script™ RT Master Mix Kit (TaKaRa Biotechnology, Dalian, China); detection was performed by the LightCycler® 96 system (Roche Diagnostics, Basel, Switzerland); and ChamQ™ SYBR® qPCR Master Mix (Vazyme Biotech, Nanjing, China) was applied for the experiment. Mouse glyceraldehyde 3-phosphate dehydrogenase (GAPDH) was used as internal control compared with genes of interest. Nucleotide sequences of the primers are listed in Supplementary Table 1.

### Enzyme-Linked Immunosorbent Assay

Spleen cells from mice were isolated and cultivated in RPMI 1640, added with 10% fetal bovine serum (Corning), and activated with 25 μg/ml of MOG_35−55_ for 72 h. Supernatants were harvested and determined for IFN-γ, IL-17A, GM-CSF, IL-1β, IL-5, IL-6, and IL-10 by ELISA Kits (R&D Systems, Minneapolis, MN, USA).

### Flow Cytometry

For cell surface staining, fluorochrome-conjugated Abs to CD4/CD8 (BD Biosciences, San Jose, CA, USA) or isotype Abs were incubated with cells for 0.5 h on ice. For intracellular cell staining, CNS-penetrated MNCs or splenocytes were stimulated with phorbol 12-myristate 13-acetate (PMA; 50 ng/ml), ionomycin (500 ng/ml) (Sigma-Aldrich), and GolgiPlug (BD Biosciences) for 5 h. The staining process was carried out following our earlier method (15). Details of all flow cytometry antibodies used are listed in Supplementary Table 2. Results were evaluated by FlowJo 10.4 (Tree Star, Ashland, OR, USA).

### Statistical Analysis

Statistical analyses were carried out by GraphPad Prism 8 (GraphPad, La Jolla, CA, USA). Results are provided as mean ± SD. All data were analyzed by the Mann–Whitney test and ANOVA with Tukey's multiple comparisons test. Statistical details are given in the figure legends. Differences with *p*-values of < 0.05 were considered significant.

## Results

### Silybin Effectively Alleviates Clinical Experimental Autoimmune Encephalomyelitis

**Figure 1:**
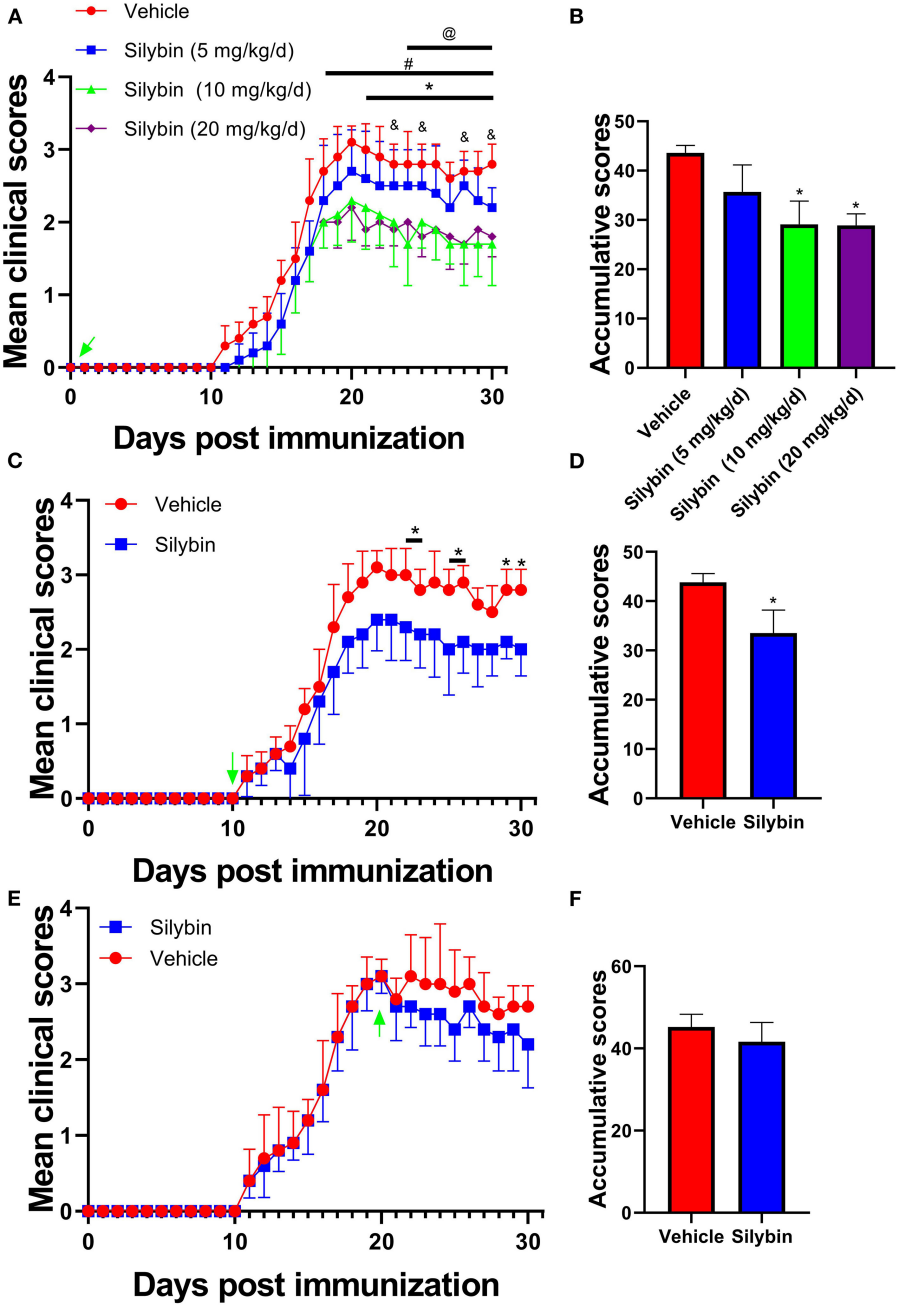
Silybin alleviated the induction of experimental autoimmune encephalomyelitis (EAE). **(A)** Clinical scores were recorded daily for i.p. injection of silybin (5, 10, 20 and mg/kg/day) to EAE mice from the time of disease induction (0 day p.i.). *, comparison between 10 mg/kg/day and vehicle; #, comparison between 20 mg/kg/day and vehicle; @, comparison between 10 and 5 mg/kg/day; &, comparison between 20 and 5 mg/kg/day. **(C)** Clinical scores of mice treated by daily i.p. injection of silybin (10 mg/kg/day) or vehicle alone, beginning at the time of disease onset (10 day p.i.). **(E)** Clinical scores of mice treated by daily i.p. injection of silybin (10 mg/kg/day) or vehicle alone, starting at the time of peak stage of the disease (20 day p.i.) and scored daily following a 0–5 scale. **(B,D,F)** Accumulative score of EAE (sum of daily clinical scores from disease onset). Data are shown as mean values ± SD (*n* = 5 each group). Two-way ANOVA or nonparametric Mann–Whitney test was used. ^*^p < 0.05, one representative of three independent experiments is shown.

To evaluate the anti-inflammatory properties of silybin, we determined its therapeutic ability in EAE. To test various doses, we noticed that silybin at 10 and 20 mg/kg/day is optimal for alleviating EAE development (*p* < 0.05; Figures 1A,B). In order to reduce the unmet effects, a lower dosage of 10 mg/kg/day was used for a later *in vivo* test. To dissect the ability of silybin in EAE, treatment began on day 10 p.i., when pathogenic T cells had begun migrating to the CNS (16). The silybin-treated EAE group showed decreased disease development than did the vehicle-treated group (Figures 1C,D). These data indicated that silybin might also prevent pathological inflammatory cells from aggravating the disease status. In addition, silybin treatment begins from the peak period, which was also tested in this study. The results showed that silybin failed to relieve the severity of the disease's progression (Figures 1E,F).

**Figure 2:**
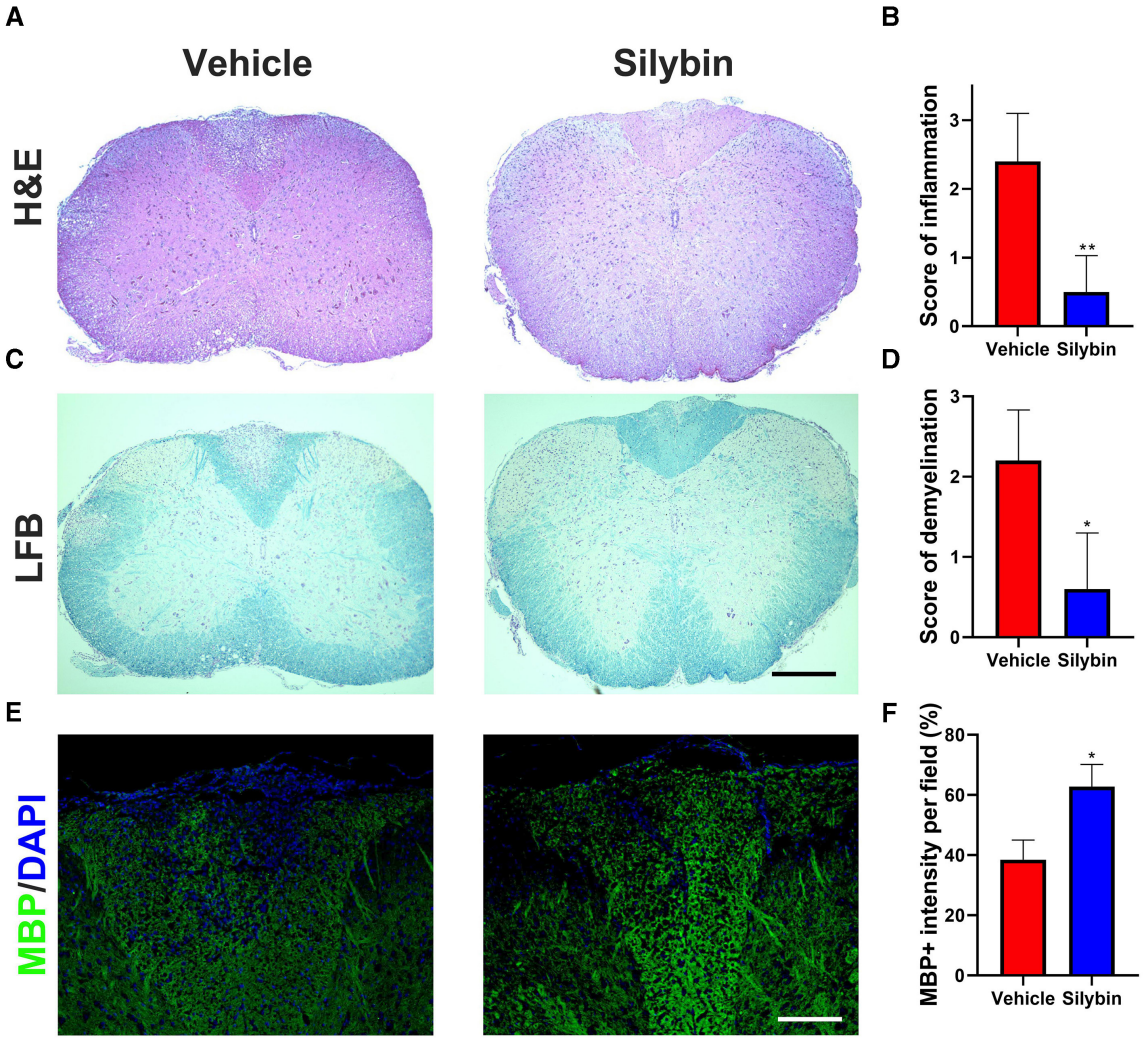
Silybin treatment ameliorated the inflammatory cell infiltration and demyelination. **(A,C)** Mice were treated with silybin (10 mg/kg/day) from day 0 and sacrificed on day 21 p.i. (*n* = 5 each group), and spinal cords (SCs) were harvested for H&E and luxol fast blue (LFB) staining. The white matter of the lumbar SC was analyzed to assess inflammation and demyelination. **(B,D)** Mean scores of inflammation in H&E-stained and demyelination in LFB-stained spinal cord sections. **(E)** Sections were assayed for myelinated area by myelin basic protein (MBP) staining. **(F)** Quantitative analysis of MBP expression. MBP intensity was measured in the lesion areas in the SCs using Image-Pro. Data represent mean ± SD (*n* = 8 each group). Scale bar = 500 μm **(A,C)** or 100 μm **(E)**. *p < 0.05 and **p < 0.01. Student's *t*-test. One representative of three independent experiments is shown.

To examine the influence of silybin on CNS pathology of EAE, SCs from silybin-treated and control groups (treated with silybin or vehicle starting from day 0 p.i. and sacrificed at day 21 p.i.) were analyzed for inflammation and demyelination. Silybin-treated EAE mice had decreased inflammation and demyelination than did controls (Figures 2A–D). Furthermore, MBP staining demonstrated that the demyelination lesion was greatly reduced in silybin-treated mice (Figures 2E,F). Higher MBP expression in the WM of the silybin-treated group indicates that silybin may prevent demyelination via suppressing inflammatory cell activation or migration.

### Silybin Administration Effectively Reduces the Peripheral Immune Response

**Figure 3:**
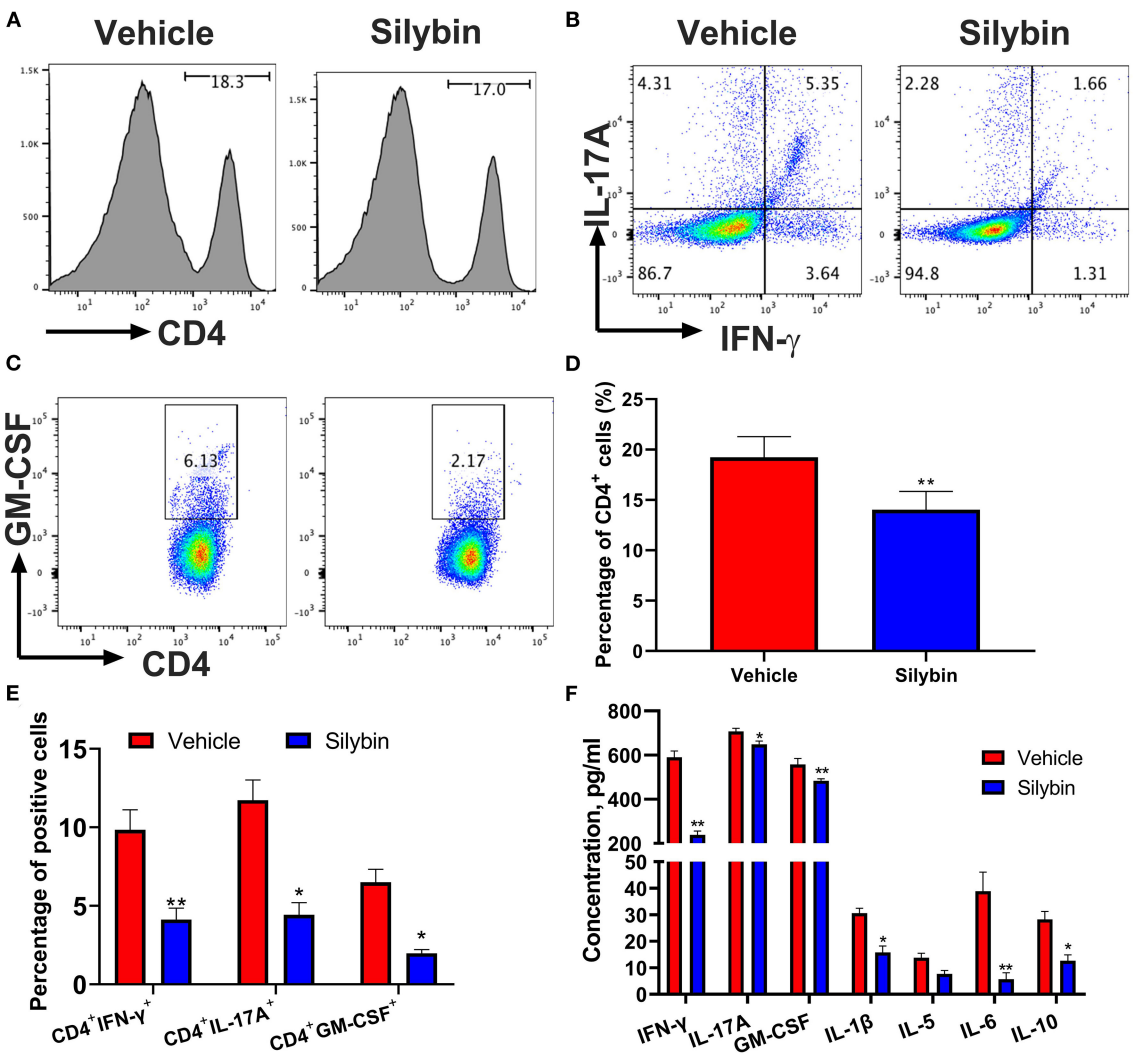
Silybin treatment suppressed inflammatory response in the periphery. mononuclear cells (MNCs) of the spleen harvested from experimental autoimmune encephalomyelitis (EAE) mice described in Figure 1A (10 mg/kg/day) and isolated (*n* = 3 mice each group) at the day of 21 p.i. **(A)** The percentage of CD4^+^ in lymphocyte gate of the above mice was analyzed by flow cytometry. Percentages of positive cells for these markers in the periphery are expressed as mean ± SD. **(B,C)** Subsets of Th1, Th17, and GM-CSF^+^ cells in CD4^+^ gate were analyzed by intracellular staining of IFN-γ^+^, IL-17A^+^, and GM-CSF^+^ from the periphery. Percentages of positive cells for **(D)** CD4^+^ and CD8^+^ and **(E)** Th1, Th17, and GM-CSF^+^CD4^+^ T cells in the periphery are expressed as mean ± SD. **(F)** Supernatants derived from splenocyte cultures described in the section Material and Methods were analyzed for the level of indicated cytokines. Data are mean ± SD (*n* = 3). Statistical significance was determined by unpaired Student's *t*-test (*p < 0.05 and **p < 0.01).

In the following, the immunomodulatory activities of silybin were assessed. Spleen cells were collected at 21 days p.i., stimulated with MOG_35−55_ (25 μg/ml) for 3 days *ex vivo*, and determined by flow cytometry. In comparison with the vehicle-treated group, CD4^+^ cells in the silybin treatment group were lower. However, no significant difference was observed between these two groups (Figures 3A,D). Furthermore, CD4^+^IFN-γ^+^ (Th1), CD4^+^IL17^+^ (Th17), and CD4^+^GM-CSF^+^ cells (Figures 3B,C,E) were examined in the spleen of the silybin-treated group. The gating strategies are shown in Supplementary Figure 1. Supernatants of spleen cells were determined by ELISA to test the activities of silybin on MOG-stimulated cytokine production. The pro-inflammatory molecules of IFN-γ, IL-1β, and IL-6 were greatly decreased in the silybin-treated mice. Compared with that in the vehicle-treated group, the IL-17A concentration in the silybin-treated group was significantly reduced, and GM-CSF also showed a similar trend (Figure 3F). Therefore, we asked whether silybin affected the maturation of DC. We will verify this speculation in subsequent experiments. In addition, the IL-5 and IL-10 representing the Th2 and an anti-inflammatory cytokine were also reduced after silybin administration. Taken together, these data suggested that silybin weakened the disease progression of EAE by comprehensively inhibiting immune cell activation, especially Th1 and Th17 differentiation *in vivo*.

### Silybin Treatment Suppresses Central Nervous System Inflammatory Infiltration

**Figure 4:**
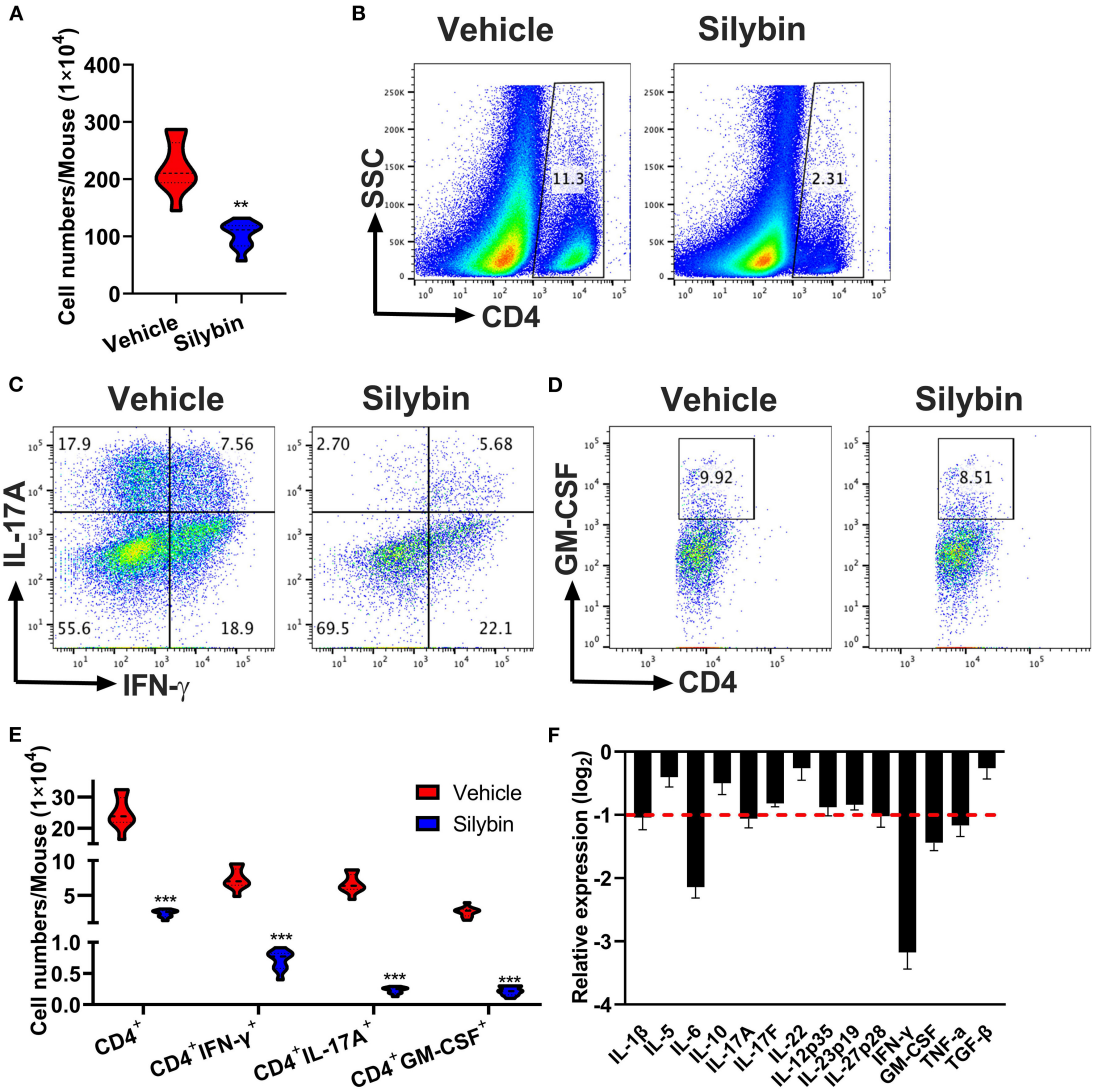
Silybin therapeutic blocked inflammatory infiltration in the central nervous system (CNS). Mice were treated with vehicle or silybin at the day of experimental autoimmune encephalomyelitis (EAE) induction and sacrificed at day 21 p.i. Spinal cords and brain were collected and mononuclear cells (MNCs) separated (*n* = 5 each group). **(A)** Total MNC numbers in CNS were counted by a light microscope. **(B)** The percentage of CD4^+^ T cells was measured by flow cytometry. **(C,D)** Frequencies of IFN-γ^+^, IL-17A^+^, and GM-CSF^+^ cells among CD4^+^ cells were assessed by flow cytometry. **(E)** Absolute numbers of infiltrated CD4^+^ T and Th subsets were calculated by multiplying the percentages of these cells with total numbers of MNCs in each CNS tissue. **(F)** The expression of cytokine genes was determined using real-time RT-PCR analysis, and their relative expression was calculated by log_2_ of –ΔΔCt values from triplicate of PCR. More than two-fold changes (log_2_ < −1) were considered significant between groups (red dotted line). Symbols represent mean ± SD (*n* = 5 each group). **p < 0.01 and ***p < 0.001. Student's *t*-test. One representative of three independent experiments is shown.

To determine the treatment outcome of silybin on CNS pathology, MNCs were separated from CNS and measured by flow cytometry. The whole number of MNCs was 220.6 ± 45.17 × 10^4^ cells/mouse in the vehicle-treated group compared with 102.8 ± 22.95 × 10^4^ in the silybin-treated group (*p* < 0.001, Figure 4A). Fewer numbers of penetrating CD4^+^ T cells, Th1, Th17, and GM-CSF^+^CD4^+^ T cells were observed in the CNS of silybin-treated mice compared with the vehicle-treated mice (Figures 4B–E). The gating strategies are shown in Supplementary Figure 2. Furthermore, to clarify how silybin administration suppressed inflammatory cell response, we determined the inflammatory molecules' expression level in the SC of vehicle- and silybin-treated mice. As shown in Figure 4F, expression of inflammatory cytokine, comprising IFN-γ, IL-1β, IL-6, IL-17A, and GM-CSF, significantly decreased in mice treated with silybin. These results demonstrated that silybin considerably blocks inflammatory cell response and infiltration in the CNS.

### Silybin Inhibits Bone Marrow-Derived Dendritic Cell Activation *in vitro*

**Figure 5:**
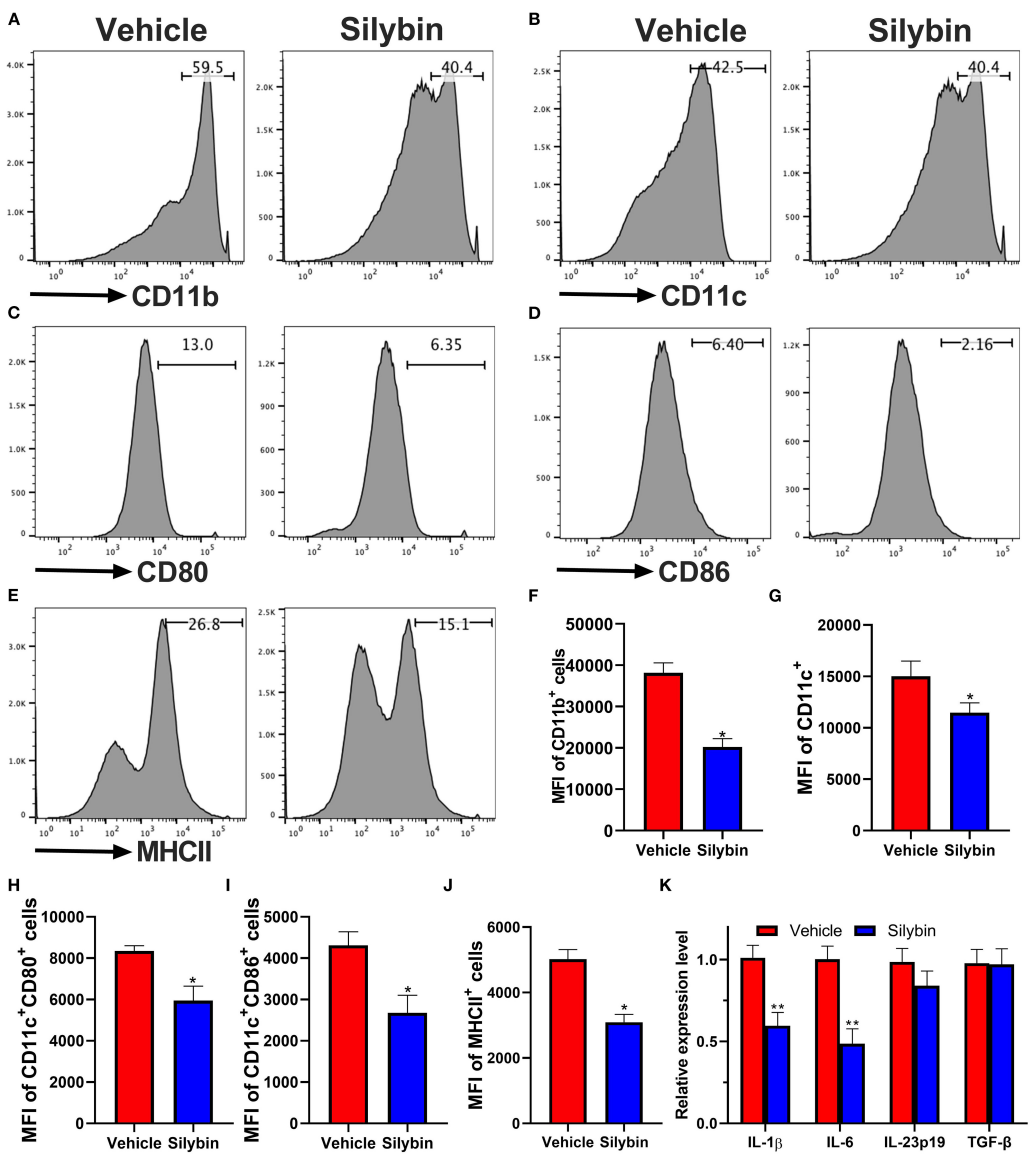
Silybin inhibited the activation of bone marrow-derived dendritic cells (BM-DCs) *in vitro*. BM-DCs were generated and stimulated with 100 ng/μl of lipopolysaccharide (LPS), and simultaneously, silybin at a dose of 50 μM was added into the culture medium. After 18 h, expression of **(A)** CD11b, **(B)** CD11c, **(C)** CD80, **(D)** CD86, and **(E)** MHC II (the positive cells of CD80, CD86, and MHC II are gated below the CD11c-positive cells) was measured by flow cytometry following overnight incubation and treatment with or without silybin. **(F–J)** Percentages of each molecule were counted. **(K)** Figures **(A–E)** are representatives of three independent experiments. Statistical data are expressed as mean ± SD of three independent experiments. *p < 0.05 and **p < 0.01 by Student's *t*-test.

Because the silybin possessed the most significant therapeutic effect in the prophylactic disease stage, and ELISA and RT-PCR data show that the expression of IL-1β and IL-6 is remarkably downregulated, we speculate whether silybin has a therapeutic effect on DCs. Here, we tested its direct effects on DC activation in culture. For this reason, BM-DCs were extracted and cultivated. DCs were stimulated by LPS and expressed relatively high levels of CD11b, CD11c, CD80, CD86, and MHC class II markers of DC activation; and the level of these molecules was greatly suppressed by silybin administration (Figures 5A–J). The gating strategies are shown in Supplementary Figure 3.

To further examine the inhibitory effect of silybin on BM-DC activation, we then determined the mRNA levels of multiple inflammatory-associated genes expressed by BM-DCs. Kim et al. have reported that silybin has a significant inhibitory effect on Th1 cells (17) but that its effect on Th17 is unknown. Previous studies showed that IL-1β, IL-6, IL-23, and TGF-β are crucial to the Th17 differentiation (18). In this study, we focused on testing the effect of silybin on these cytokines. The data showed that silybin mainly inhibited the expression of IL-1β and IL-6. It is suggested that silybin may indirectly block the polarization of Th17 cells by regulating the activity of DCs (Figure 5K).

### Silybin Treatment Blocks T-Cell Proliferation and Polarization

To study *ex vivo* proliferation response affected by autoantigen MOG_35−55_ in splenocytes of the vehicle and silybin-treated mice starting from day 0 p.i., the mice were euthanized on day 21 p.i., and low proliferation efficiency was observed in both vehicle- and silybin-treated groups without antigen stimulation. While cells were pulsed with MOG_35−55_, a stronger response was recorded in the vehicle-treated group compared with the silybin-treated group. By contrast, no significant difference was observed in both vehicle- and silybin-treated groups, which responded to Con A stimulation (Supplementary Figure 4). This result indicated that silybin administration suppressed the MOG_35−55_-induced proliferation response.

**Figure 6:**
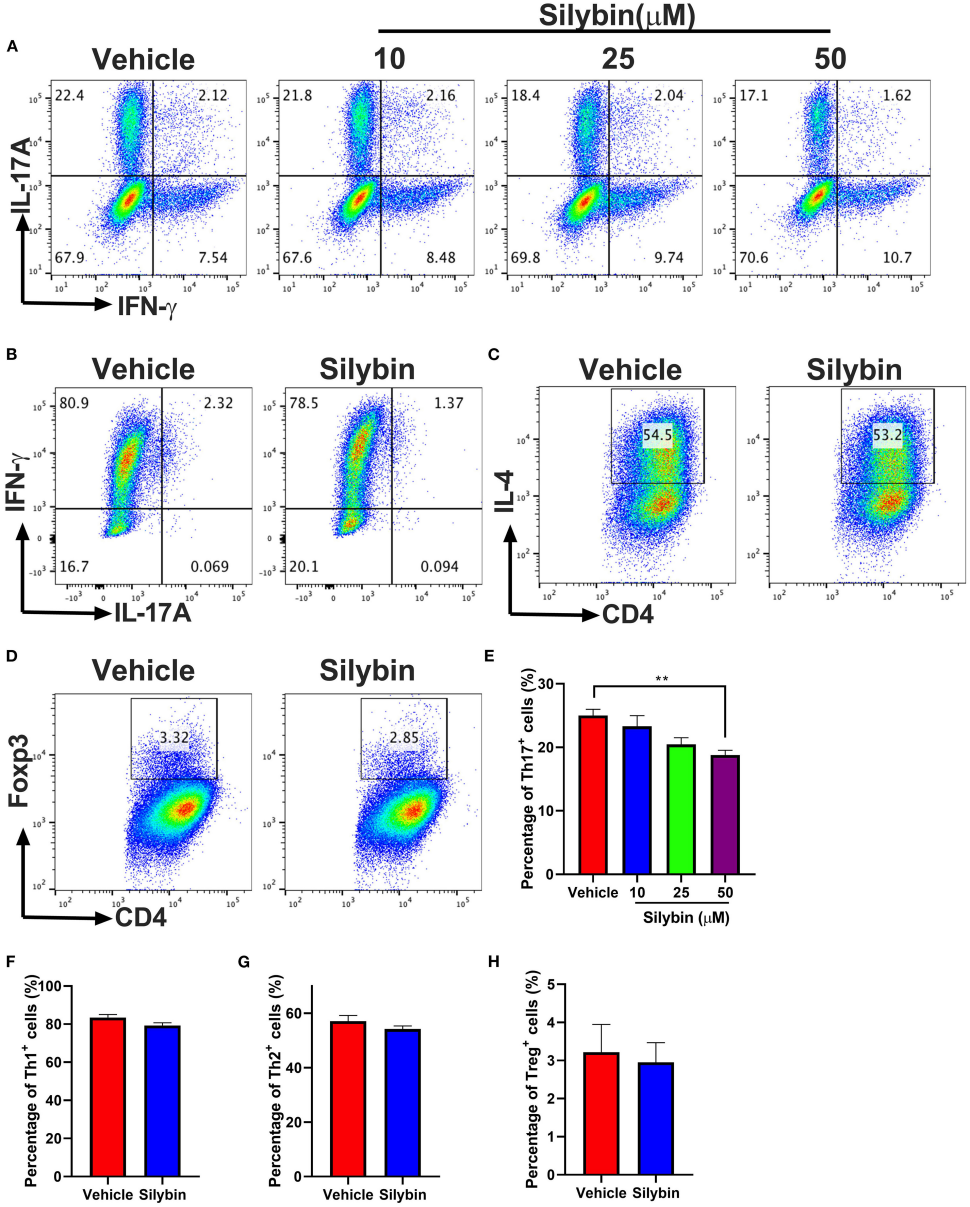
Silybin inhibited Th cell subset differentiation. **(A)** CD4^+^ cells were isolated from mice and cultured under the Th17-polarizing condition with different concentrations of silybin for 3 days. Percentage of IL-17A^+^ cells was analyzed by intracellular staining of IL-17A. **(B–D)** CD4^+^ cells were cultured under the Th1, Th2, and Treg-polarizing conditions with silybin (50 μM) for 3 days. Percentages of Th1, Th2, and Treg cells were analyzed by intracellular staining of IFN-γ^+^, IL-4^+^, and Foxp3^+^, respectively. The RNA extracted from the vehicle- or silybin-treated DCs and RT-PCR was performed to determine the mRNA expression level of IL-1β, IL-6, IL-23, and TGF-β. **(E–H)** Statistical analysis of **(A–D)**. Data represent mean ± SD (*n* = 3 each group). **p < 0.01. Student's *t*-test. One representative of three independent experiments is shown.

To elucidate the mechanism underlying the activities of silybin on various Th17 cell populations, the differentiation efficiency of T-cell subsets was tested. Under Th17-polarization media, approximately 25% of cells were IL-17A positive in the vehicle-treated group, though silybin (50 μM) exhibited great activity to decrease IL-17A expression by T cells (24.80 ± 1.253% vs. 18.66 ± 0.794%, *p* < 0.01) (Figures 6A,E). We then studied the activities of silybin on Th1, Th2, and Treg cell polarization. Unlike the Th17 cell differentiation result, IFN-γ, IL-4, and Foxp3 expression under Th1, Th2, and Treg differentiation conditions were not effectively suppressed by silybin (Figures 6B–D,F–H). The gating strategies are shown in Supplementary Figure 5. Interestingly, although the expression of IFN-γ increased slightly under the Th17 polarization condition in the silybin-treated group, no significant difference was observed compared with the vehicle-treated group (Supplementary Figure 6A). Also, we found that silybin only specifically suppressed ROR-γt expression in CD4^+^ T cells under Th17-polarizing conditions. In contrast, T-bet, Gata-3, and Foxp3 expression in CD4^+^ T cells under Th1, Th2, or Treg cell conditions were not significantly decreased compared with those in vehicle-treated groups (Supplementary Figure 6B). Altogether, these data indicated that silybin suppressed Th17 differentiation.

### Silybin-Conditioned Bone Marrow-Derived Dendritic Cells Have a Reduced Ability to Initiate Th1 and Th17 Polarization

**Figure 7:**
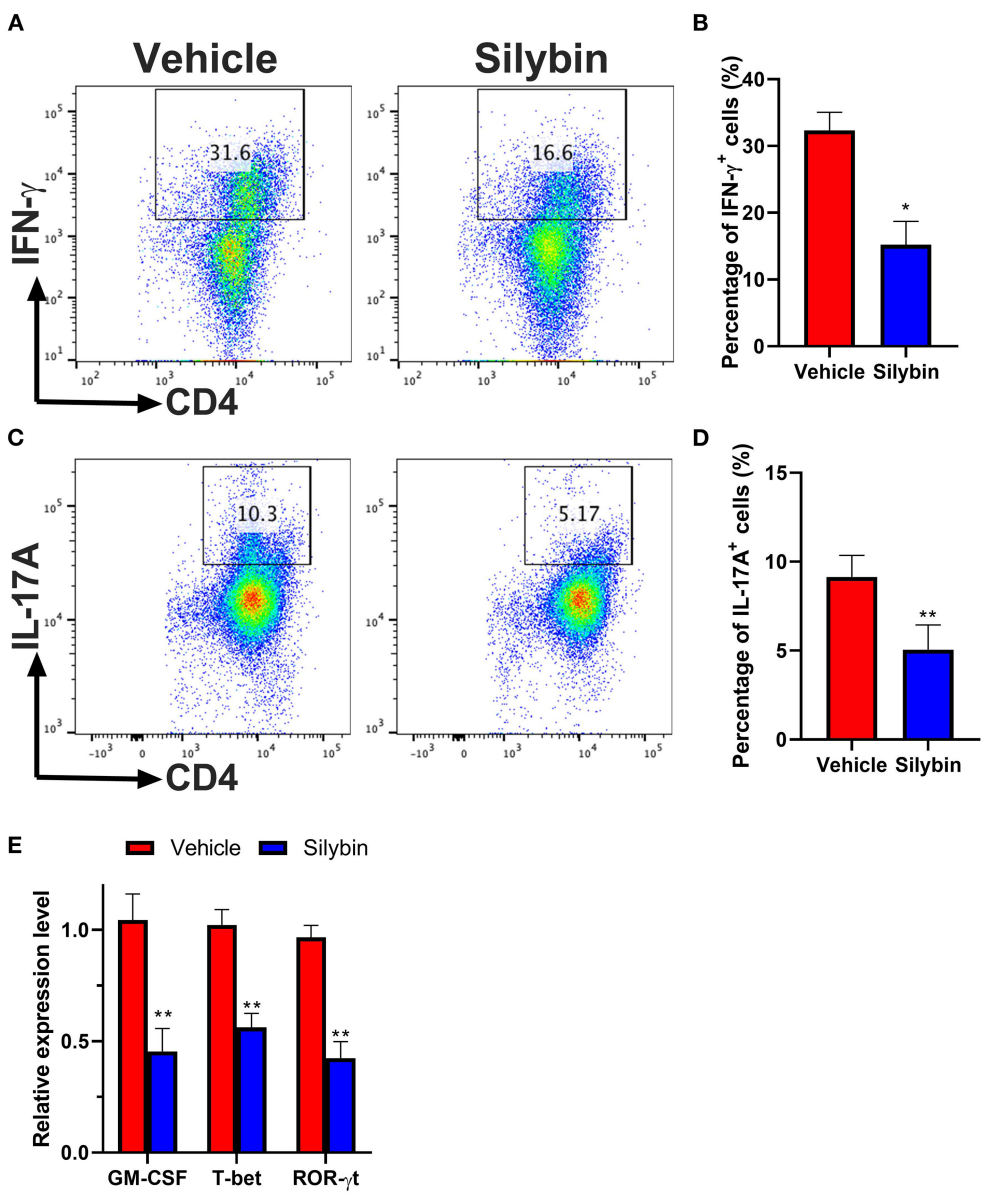
Silybin-treated bone marrow-derived dendritic cells (BM-DCs) induce Th1 and Th17 CD4+ T-cell polarization *in vitro*. **(A,C)** Intracellular staining for IFN-γ and IL-17A of naïve CD4^+^ T cells after 3 days of co-culture with lipopolysaccharide (LPS)-activated BM-DCs. **(B,D)** Analysis of IFN-γ- and IL-17A-positive CD4^+^ T cells by division number as assessed by intracellular staining. **(E)** RNA extraction of from the vehicle- or silybin-treated T cells and RT-PCR was performed to determine the mRNA expression level of granulocyte/macrophage colony-stimulating factor (GM-CSF), T-bet, and ROR-γt. Statistical data are expressed as mean ± SD of three independent experiments. *p < 0.05 and **p < 0.01 by paired *t*-test.

Then, we investigated whether BM-DCs treated by silybin inhibited the differentiation of T cells. We tried to determine whether the differential expression of MHC II and other costimulatory molecules affected the polarization of Th17. Comparison of surface marker expression of silybin- and vehicle-treated DC demonstrated apparent differences in expression of MHC II, the costimulatory molecule CD80 or CD86, indicating that there are underlying differences between the activating signals to T cells. Therefore, we conducted a co-culture test of DC and T cells. After DC was treated with silybin, the activation of T cells and the differentiation tendency of Th1 and Th17 were inhibited (Figures 7A–D). In addition, we found that silybin suppressed GM-CSF and ROR-γt expression level but not T-bet from the T cell, which was polarized by MOG_35−55_-pulsed DCs (Figure 7E). Based on this result, we speculate that silybin inhibits the polarization of Th1 mainly by blocking the activity of DC, which is different from inhibiting the polarization of Th17. This result indicates that silybin can indirectly inhibit the differentiation of T cells and affect the polarization of T cells by regulating DC.

## Discussion

In this study, we demonstrate the promising effects of silybin on both prophylactic and onset phases of EAE. Silybin blocks the migration of inflammatory cells into the CNS and inhibits the myelin damage process remarkably, thus relieving the disorder development. The function of silybin on EAE is mainly due to its repressive effects on Th17 cell polarization. Furthermore, silybin suppresses the T-cell polarization, which is dependent on the inhibition of the activation of DCs, a critical underlying mechanism of silybin for the treatment of autoimmune disease.

Silybin is a flavonoid, a primary component of silymarin, extracted from the seed of species derived from *S. marianum* (19). *S. marianum* has been used in traditional medicine for many years. In China, owing to its specific characteristics in treating liver diseases, it has been widely used for more than 2,000 years. Ancient herbalists described silybin as possessing nephron-, neuro-, hepato-, and cardio-protective activities due to its antioxidant, anti-inflammatory, and regenerative effects (20, 21). In recent years, the antioxidant activity of silybin has been reported. It can directly act on the scavenging of free radicals and block the specific enzyme generators of free radicals. Moreover, it induces non-enzymatic antioxidant defenses, such as glutathione or transcription factors (Nrf2 and NF-κB) (22, 23). Studies have shown that silybin treatment attenuates the production of prostaglandin E2, IL-1β, and major chemotactic protein-1 (MCP-1), suggesting that silybin has a significant anti-inflammatory effect via suppressed NF-κB activity (24). Based on this theory, Min et al. used silybin to treat EAE mice and found that it can inhibit the disease development significantly (25). These results are consistent with our conclusion and encourage us to identify the possible mechanism of action; we found the inhibitory effects of silybin on DC activation in an inflammatory state. Moreover, the results also showed that silybin possessed significant inhibitory activity on Th17 differentiation. However, there are also some conflicting conclusions. For example, we found that silybin does not exhibit a comprehensive suppression effect and immunomodulatory activity on the immune response, because silybin does not alter the differentiation efficiency of Th2 and Treg cells significantly. For autoimmune diseases, candidate drugs can usually show a significant inhibitory effect on autoreactive T cells and can exhibit a certain immunomodulatory effect. However, in this study, our results showed that silybin not only inhibited pro-inflammatory cytokines but also blocked the production of anti-inflammatory cytokines. This may be related to the use dosage of silybin, and it will need further test in the future experiments. We have also noticed that the differences in some details of the experiment may lead to the opposite results, such as the amount of PT injection, the manner of drug administration, and the sacrifice time of mice.

Previous studies reported that silybin also possessed tissue regeneration functions. Tabandeh et al. found that silybin treatment could increase stromelysin-1 expression and extracellular matrix constituents, thus promoting the wound healing process (26). Silybin complex with copper(II) ion stimulates the expression of osteoblastic marker genes, such as Runx2, ALP, type 1 collagen, and OCN at the molecular level, and enhances osteoblast differentiation (27). In addition, in Alzheimer's disease, silybin-treated APP/PS1 transgenic mice showed higher numbers of newly generated microglia, astrocytes, neurons, and neuronal precursor cells, indicating its positive effects on neuro-regeneration. Based on these data, we speculate whether silybin also has functions for the treatment of the CNS demyelinating diseases. We tested its effects on the differentiation of oligodendrocytes, but no significant differences were observed (data not shown). In another study, Tsai et al. showed that silymarin has a better protective effect than silybin in the SC injury model. It is indicated that the regenerative function might be an indirect effect, which is achieved through the inhibition of peroxide-induced reactive oxygen species (ROS) (28).

So far, there are few reports on the effects of silybin on DCs. As early as 2007, Lee et al., for the first time, reported silybin on the phenotypic and functional maturation of murine BM-derived DCs. Silybin was shown to strongly inhibit CD80, CD86, MHC class I, and MHC class II expression on the surface of DCs and was also related to the impairments of LPS-induced IL-12 expression of DCs (17). In this study, based on previous work, we further tested the activity of silybin on DC activation in the EAE model. The co-culture system of DCs and T cells was employed to test that the inhibitory effects of silybin on Th1 and Th17 differentiation. The results indicated that the inhibiting effects of silybin are partially dependent on the DCs.

A number of studies have proved that silybin significantly promoted T-cell activation and proliferation (8, 25). In other words, silybin has also exhibited immunomodulatory effects (6). However, in our study, we did not observe that silybin treatment significantly altered the percentage of Th1, Th2, and Treg. Similar to the previous results, silybin did not significantly affect Th2 cells (25). In addition, although Min's research showed that silybin inhibited Th1-related cytokine production, such as IL-2 and IL-12, Min did not test the expression of IFN-γ (25). In another study, Lee et al. showed that silybin inhibited the polarization of Th1 cells. Nevertheless, this result comes from LPS-treated chronic inflammation mice with distinct pathogenesis of MOG_35−55_-induced EAE mice. For the first time, our study used anti-CD3/28 and various cytokines to polarize the T subset *in vitro*, and we tested the effects of silybin on these populations. Interestingly, the polarization experiment of silybin on Th1 cells is not consistent with the results of DC and T-cell co-culture. We speculate that it may be due to the dosage of silybin. Perhaps silybin possesses immunomodulatory capabilities at low doses. This hypothesis will be further verified in our subsequent experiments.

The structure of silybin can be depicted as two sections with carbo- and heterocycles. One section of the molecule is a flavonol group, taxifolin; another is a unit of coniferyl alcohol phenylpropanoid; and they are linked by an oxirane ring (29). It is highly soluble in polar aprotic solutions such as DMSO, acetone, tetrahydrofuran (THF), and *N, N*-dimethylformamide (DMF). It is hardly soluble in ethanol or methanol and is insoluble in non-polar solvents such as chloroform and petroleum ether. In addition, because the molecular weight of silybin is high, it cannot be absorbed by simple diffusion, and the oral bioavailability is low (30). Therefore, the solubility of silybin as a drug limits its therapeutic effects. At present, there are some targeted drug carriers, such as the use of nanoparticles or exosomes (31, 32), which may contribute to the clinical therapeutic effect of silybin.

In conclusion, our study demonstrated that silybin is a valuable anti-inflammatory agent to treat autoimmune disease and therapeutically manage such as MS.

## Data Availability Statement

The original contributions presented in the study are included in the article/Supplementary Material, further inquiries can be directed to the corresponding author.

## Ethics Statement

The animal study was reviewed and approved by Animal Ethics Committee of Xian Yang Central Hospital.

## Author Contributions

H-LY and X-WS conceived and designed the experiments, carried out the experiments, and wrote the manuscript. All authors read and approved the final manuscript.

## Conflict of Interest

The authors declare that the research was conducted in the absence of any commercial or financial relationships that could be construed as a potential conflict of interest.

## Publisher's Note

All claims expressed in this article are solely those of the authors and do not necessarily represent those of their affiliated organizations, or those of the publisher, the editors and the reviewers. Any product that may be evaluated in this article, or claim that may be made by its manufacturer, is not guaranteed or endorsed by the publisher.
