# Supplementary material for: Silybin Alleviates Experimental Autoimmune Encephalomyelitis by Suppressing Dendritic Cell Activation and Th17 Cell Differentiation
Source: Front Neurol. 2021 Sep 7;12:659678. doi: 10.3389/fneur.2021.659678 (PMC8452861; doi:10.3389/fneur.2021.659678)
Supplement: Supplementary file 1 [file Table_1.DOCX]

**Supplementary Information**

Supplementary Figure 1. Gating strategies of (a) Th1 and Th17 and (b)CD4^+^GM-CSF^+^ cells in *ex vivo* experiments from spleen.

Supplementary Figure 2. Gating strategies of (a) Th1 and Th17 and (b)CD4^+^GM-CSF^+^ cells in *ex vivo* experiments from CNS monocytes.

Supplementary Figure 3. Gating strategies of microglia and dendritic cells.

Supplementary Figure 4. Effect of glucosamine on proliferative responses *ex vivo*.

Supplementary Figure 5. Gating strategies of (a) Th17, (b)Th1, (c)Th2, and (d)Treg cells in *in vitro* experiments.

Supplementary Figure 6. The modulation effect of silybin on IFN and transcription factors.

Sup Table1. Primers used for real-time quantitative RT-PCR analysis

Sup Table2. Flow cytometry antibody list


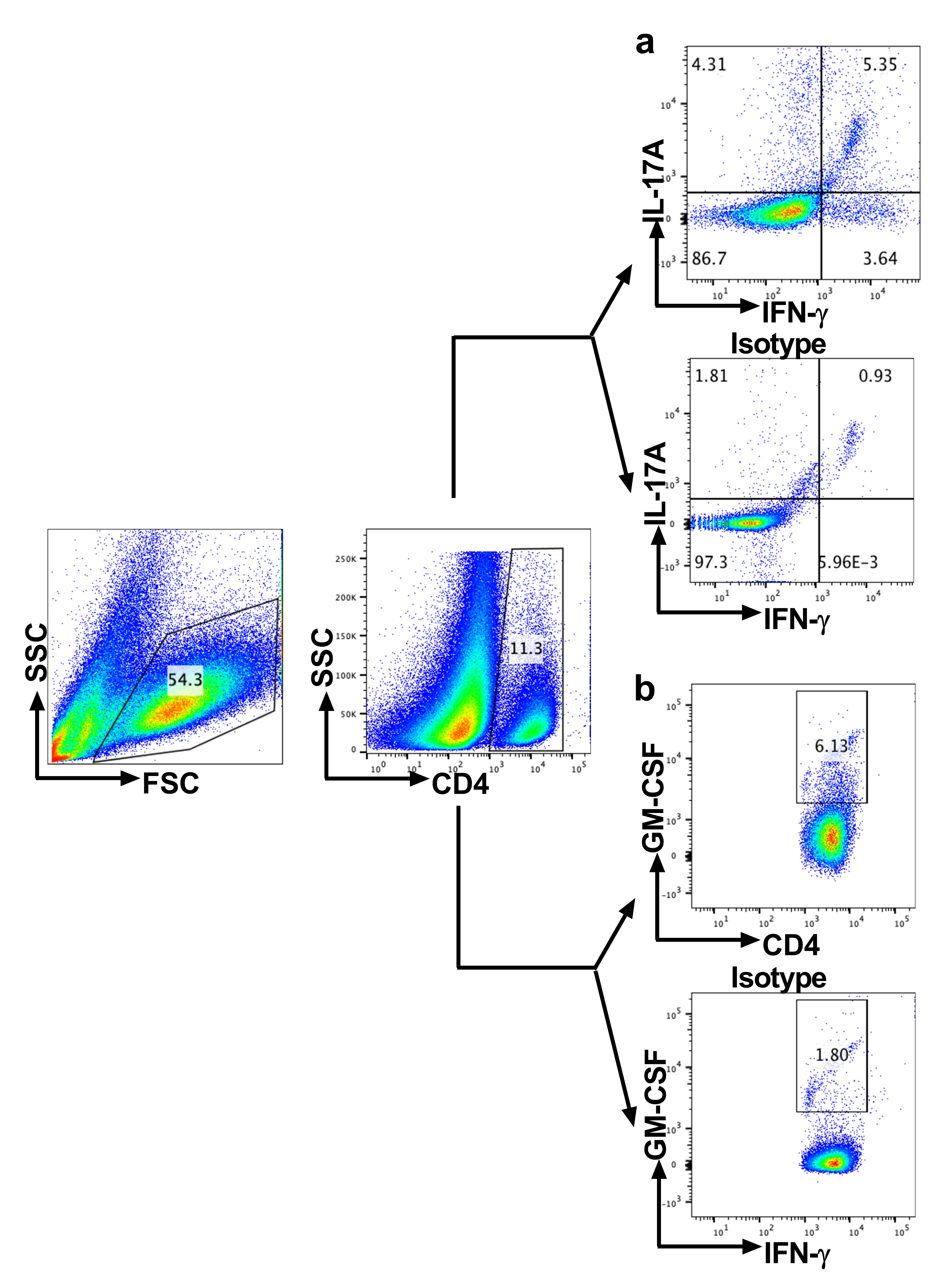


**Supplementary Figure 1. Gating strategies of (a) Th1 and Th17 and (b)CD4^+^GM-CSF^+^ cells in *ex vivo* experiments from spleen.** Cells were stained by Abs to CD4 surface-marker, IFN-γ, IL-17A, and GM-CSF intercellular-marker or isotype control Abs for 30 min on ice. Negative cells were excluded by isotype control and every cell subset was analyzed by FlowJo software.

**
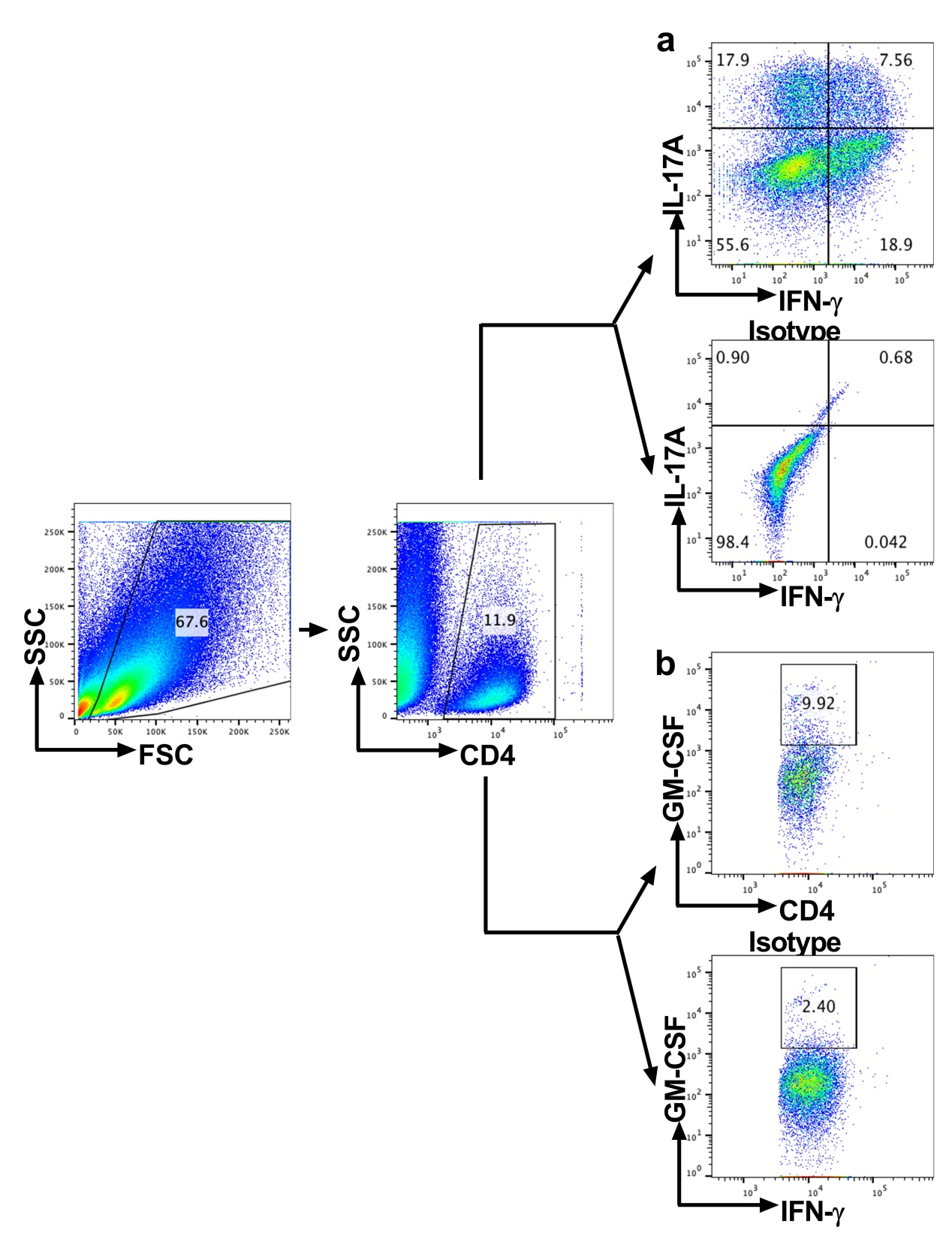
**

**Supplementary Figure 2. Gating strategies of (a) Th1 and Th17 and (b)CD4^+^GM-CSF^+^ cells in *ex vivo* experiments from CNS monocytes.** Cells were stained by Abs to CD4 surface-marker, IFN-γ, IL-17A, and GM-CSF intercellular-marker or isotype control Abs for 30 min on ice. Negative cells were excluded by isotype control and every cell subset was analyzed by FlowJo software.


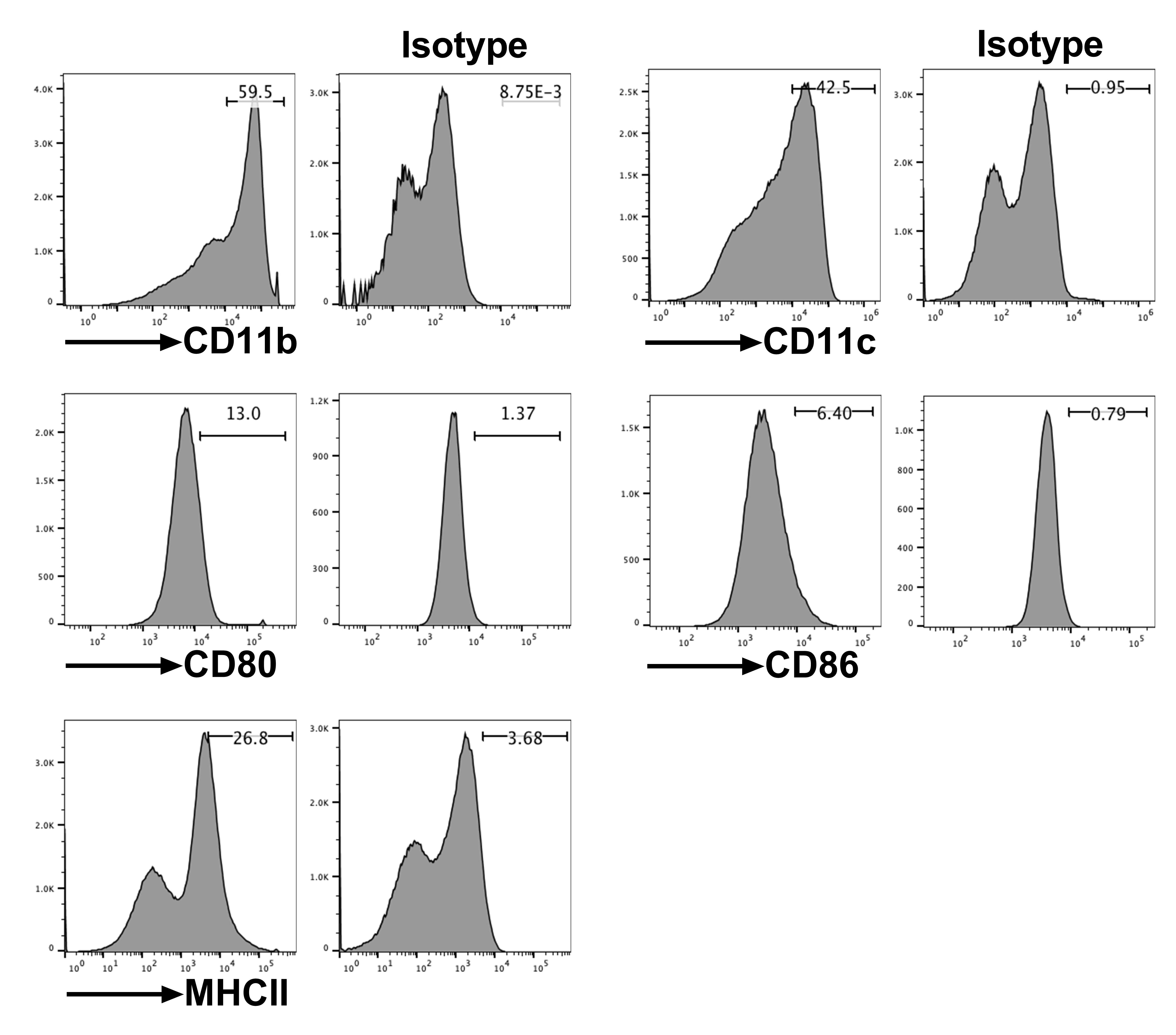


**Supplementary Figure 3. Gating strategies of microglia and dendritic cells.** Cells were stained by Abs to CD11b, CD11c, CD80, CD86, and MHCII surface-marker or isotype control Abs for 30 min on ice. Negative cells were excluded by isotype control and every cell subset was analyzed by FlowJo software.

**Supplementary Figure 4. Effect of glucosamine on proliferative responses *ex vivo*.** At day 21 p.i., splenocytes of vehicle- and silybin-treated EAE mice starting from day 0 p.i. were harvested (n = 5 in each group). A total of 4× 10^5^ splenocytes/200 μl was cultured with MOG_35–55_ at 25 μg/ml, Con A at 5 μg/ml, and without antigeng/mitogen (Ag) for proliferative response. Bars represent mean values ± SD. Values of prefer to comparison between PBS-i.p. and glucosamine-i.p. groups. **, *p* < 0.01. One representative experiment of three is shown.


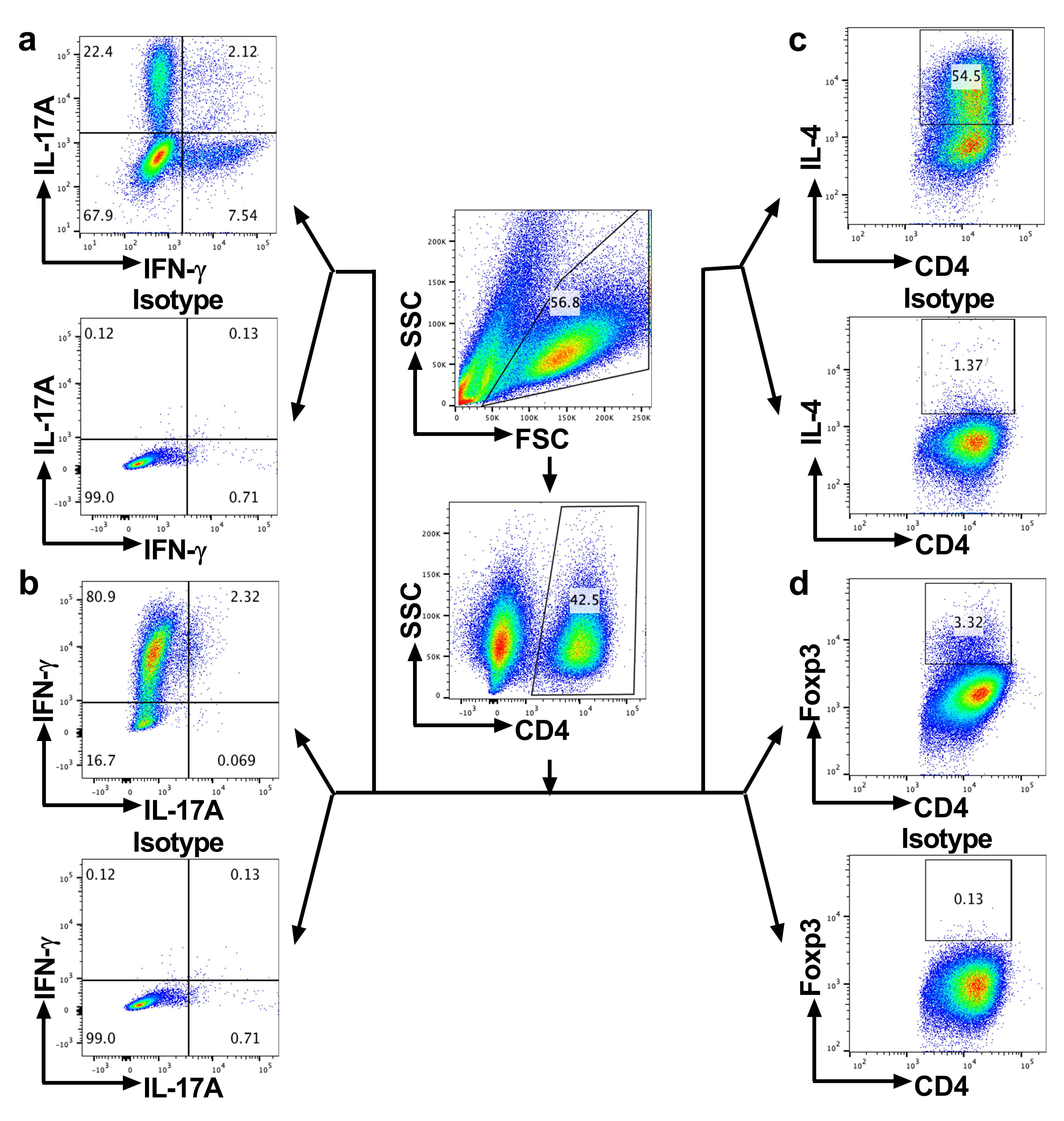


**Supplementary Figure 5. Gating strategies of (a) Th17, (b)Th1, (c)Th2, and (d)Treg cells in *in vitro* experiments.** Cells were stained by Abs to CD4 surface-marker, IFN-γ, IL-4, IL-17A, and Foxp3 intercellular-marker or isotype control Abs for 30 min on ice. Negative cells were excluded by isotype control and every cell subset was analyzed by FlowJo software.

**Supplementary Figure 6. The modulation effect of silybin on IFN and transcription factors.** (a) CD4^+^ cells were isolated from mice and cultured under the Th17 polarizing condition with different concentrations of silybin for 3 days. Percentage of IFN-γ^+^ cells was analyzed by intracellular staining of IFN-γ. (B) CD4^+^ cells were cultured under the Th1, Th2, Th17, and Treg polarizing condition with silybin (50 µM) for 3 days. The relative mRNA expression level of T-bet, Gata-3, ROR-γt, and Foxp3 were determined by RT-PCR., respectively. Statistical data are expressed as mean ± SD of three independent experiments. **p < 0.01 by pair t-test.

**Sup Table1. Primers used for real-time quantitative RT-PCR analysis**

| Gene | Forward | Rewards | |
| --- | --- | --- | --- |
| IL-1β | CTCTCCACCTCAATGGACAGA | | TGCTTGGGATCCACACTCTC |
| IL-5 | TGTCCCTACTCATAAAAATCACCAG | | TCCGTCTCTCCTCGCCACAC |
| IL-6 | ACACATGTTCTCTGGGAAATCGT | | AAGTGCATCATCGTTGTTCATACA |
| IL-10 | GCTCTTACTGACTGGCATGAG | | CGCAGCTCTAGGAGCATGTG |
| IL-17A | TTTAACTCCCTTGGCGCAAAA | | CTTTCCCTCCGCATTGACAC |
| IL-17F | TGCTACTGTTGATGTTGGGAC | | AATGCCCTGGTTTTGGTTGAA |
| IL-22 | GTGAGAAGCTAACGTCCATC | | GTCTACCTCTGGTCTCATGG |
| IL-12p35 | CATCGATGAGCTGATGCAGT | | CAGATAGCCCATCACCCTGT |
| IL-23p19  IL-27p28  IFN-γ  GM-CSF  TNF-α  TGF-β  T-bet  Gata-3  ROR-γt  Foxp3 | GACTCAGCCAACTCCTCCAG  CAGATAGCCCATCACCCTGT  ATGAACGCTACACACTGCATC  GTGGTCTACAGCCTCTCAGCA  GACGTGGAACTGGCAGAAGAG  CACTGATACGCCTGAGTG  ATTGGTTGGAGAGGAAGCGG  GGAGTCTCCAAGTGTGCGAA  CATCTCTGCAAGACTCATCG  AGGAGCCGCAAGCTAAAAGC | | GGCACTAAGGGCTCAGTCAG  GGGGCAGCTTCTTTTCTTCT  CCATCCTTTTGCCAGTTCCTC  GCATGTCATCCAGGAGGTTC  GCCACAAGCAGGAATGAGAAG  GTGAGCGCTGAATCGAAA  GCACCAGGTTCGTGACTGTA  TGGAATGCAGACACCACCTC  CAGGGGATTCAACATCAGTG  TGCCTTCGTGCCCACTGT |

**Sup Table2. Flow cytometry antibody list**

| Antibody | Color | clone | CAS | Vendor |
| --- | --- | --- | --- | --- |
| Rat Anti-Mouse CD11b | PE | M1/70 | 557397 | BD |
| Armenian Hamster Anti-Mouse CD11c | BV421 | N418 | 565452 | BD |
| Hamster Anti-Mouse CD80 | PerCP-Cy5.5 | 16-10A1 | 560526 | BD |
| Rat Anti-Mouse CD86 | APC | GL1 | 558703 | BD |
| Rat Anti-Mouse MHC II | FITC | 2G9 | 553623 | BD |
| Rat Anti-Mouse CD4 | APC | RM4-5 | 553051 | BD |
| Rat Anti-Mouse IL-4 | PE-Cy7 | 11B11 | 560699 | BD |
| Anti-Mo/Rt IL-17A | PE | eBio17B7 | 12-7177-81 | Invitrogen |
| Rat Anti-Mouse IFN-γ | BV421 | XMG1.2 | 563376 | BD |
| Rat Anti-Mo/Rt Foxp3 | FITC | FJK-16s | 11-5773-82 | Invitrogen |
| Rat Anti-Mouse GM-CSF | FITC | MP1-22E9 | 11-7331-82 | Invitrogen |
| Rat IgG2b,k Isotype Control | APC | A95-1 | 556924 | BD |
| Mouse IgG2a k Isotype Control | APC | G155-178 | 552893 | BD |
| Rat IgG2a,k Isotype Control | PerCP/Cyanine5.5 | RTK2758 | 400531 | Biolegend |
| Bat IgG2b,k Isotype Control | PE | A95-1 | 553989 | BD |
| Rat IgG2a,k Isotype Control | PE | R35-95 | 554689 | BD |
| Rat IgG2a K Iso Control | FITC | eBR2a | 11-4321-81 | Invitrogen |
| BV421 Hamster IgG1,k Isotype Control | IgG1 | A19-3 | 562601 | BD |
