# Supplementary material for: Silybin Alleviates Experimental Autoimmune Encephalomyelitis by Suppressing Dendritic Cell Activation and Th17 Cell Differentiation
Source: Front Neurol. 2021 Sep 7;12:659678. doi: 10.3389/fneur.2021.659678 (PMC8452861; doi:10.3389/fneur.2021.659678)
Supplement: Supplementary file 2 [file Table_2.DOCX]

**Supplemental information**

**Sup Table1. Primers used for real-time quantitative RT-PCR analysis**

| Gene | Forward | Rewards | |
| --- | --- | --- | --- |
| IL-1β | CTCTCCACCTCAATGGACAGA | | TGCTTGGGATCCACACTCTC |
| IL-5 | TGTCCCTACTCATAAAAATCACCAG | | TCCGTCTCTCCTCGCCACAC |
| IL-6 | ACACATGTTCTCTGGGAAATCGT | | AAGTGCATCATCGTTGTTCATACA |
| IL-10 | GCTCTTACTGACTGGCATGAG | | CGCAGCTCTAGGAGCATGTG |
| IL-17A | TTTAACTCCCTTGGCGCAAAA | | CTTTCCCTCCGCATTGACAC |
| IL-17F | TGCTACTGTTGATGTTGGGAC | | AATGCCCTGGTTTTGGTTGAA |
| IL-22 | GTGAGAAGCTAACGTCCATC | | GTCTACCTCTGGTCTCATGG |
| IL-12p35 | CATCGATGAGCTGATGCAGT | | CAGATAGCCCATCACCCTGT |
| IL-23p19  IL-27p28  IFN-γ  GM-CSF  TNF-α  TGF-β  T-bet  Gata-3  ROR-γt  Foxp3 | GACTCAGCCAACTCCTCCAG  CAGATAGCCCATCACCCTGT  ATGAACGCTACACACTGCATC  GTGGTCTACAGCCTCTCAGCA  GACGTGGAACTGGCAGAAGAG  CACTGATACGCCTGAGTG  ATTGGTTGGAGAGGAAGCGG  GGAGTCTCCAAGTGTGCGAA  CATCTCTGCAAGACTCATCG  AGGAGCCGCAAGCTAAAAGC | | GGCACTAAGGGCTCAGTCAG  GGGGCAGCTTCTTTTCTTCT  CCATCCTTTTGCCAGTTCCTC  GCATGTCATCCAGGAGGTTC  GCCACAAGCAGGAATGAGAAG  GTGAGCGCTGAATCGAAA  GCACCAGGTTCGTGACTGTA  TGGAATGCAGACACCACCTC  CAGGGGATTCAACATCAGTG  TGCCTTCGTGCCCACTGT |

**Sup Table2. Flow cytometry antibody list**

| Antibody | Color | clone | CAS | Vendor |
| --- | --- | --- | --- | --- |
| Rat Anti-Mouse CD11b | PE | M1/70 | 557397 | BD |
| Armenian Hamster Anti-Mouse CD11c | BV421 | N418 | 565452 | BD |
| Hamster Anti-Mouse CD80 | PerCP-Cy5.5 | 16-10A1 | 560526 | BD |
| Rat Anti-Mouse CD86 | APC | GL1 | 558703 | BD |
| Rat Anti-Mouse MHC II | FITC | 2G9 | 553623 | BD |
| Rat Anti-Mouse CD4 | APC | RM4-5 | 553051 | BD |
| Rat Anti-Mouse IL-4 | PE-Cy7 | 11B11 | 560699 | BD |
| Anti-Mo/Rt IL-17A | PE | eBio17B7 | 12-7177-81 | Invitrogen |
| Rat Anti-Mouse IFN-γ | BV421 | XMG1.2 | 563376 | BD |
| Rat Anti-Mo/Rt Foxp3 | FITC | FJK-16s | 11-5773-82 | Invitrogen |
| Rat Anti-Mouse GM-CSF | FITC | MP1-22E9 | 11-7331-82 | Invitrogen |
| Rat IgG2b,k Isotype Control | APC | A95-1 | 556924 | BD |
| Mouse IgG2a k Isotype Control | APC | G155-178 | 552893 | BD |
| Rat IgG2a,k Isotype Control | PerCP/Cyanine5.5 | RTK2758 | 400531 | Biolegend |
| Bat IgG2b,k Isotype Control | PE | A95-1 | 553989 | BD |
| Rat IgG2a,k Isotype Control | PE | R35-95 | 554689 | BD |
| Rat IgG2a K Iso Control | FITC | eBR2a | 11-4321-81 | Invitrogen |
| BV421 Hamster IgG1,k Isotype Control | IgG1 | A19-3 | 562601 | BD |
